# Harnessing adaptive bistable stiffness of hair-cell-bundle structure for broadband vibration applications

**DOI:** 10.1038/s41598-023-37962-9

**Published:** 2023-07-03

**Authors:** Jong-Yun Yoon, Gi-Woo Kim

**Affiliations:** 1 Department of Mechanical Engineering, Inha University, 100 Inha-ro, Michuhol-gu, Incheon, 22212 Republic of Korea; 2 Department of Mechatronics Engineering, Incheon National University, Incheon, 22012 Republic of Korea

**Keywords:** Applied physics, Sensory systems

## Abstract

This study presents an initial study on the adaptive bistable stiffness of the hair cell bundle structure in a frog cochlea, and aims to harness its bistable nonlinearity that features a negative stiffness region for broadband vibration applications such as vibration-based energy harvesters. To this end, the mathematical model for describing the bistable stiffness is first formulated based on the modeling concept of piecewise type nonlinearities. The harmonic balance method was then employed to examine the nonlinear responses of bistable oscillator, mimicking hair cells bundle structure under the frequency sweeping condition, and their dynamic behaviors induced by bistable stiffness characteristics are projected on phase diagrams, and Poincare maps concerning the bifurcation. In particular, the bifurcation mapping at the super- and sub-harmonic regimes provides a better perspective to examine the nonlinear motions which occur in the biomimetic system. The use of bistable stiffness characteristics of hair cell bundle structure in frog cochlea thus offers physical insights to harness the adaptive bistable stiffness for metamaterial-like potential engineering structures such as vibration-based energy harvester, and isolator etc.

## Introduction

**Figure 1:**
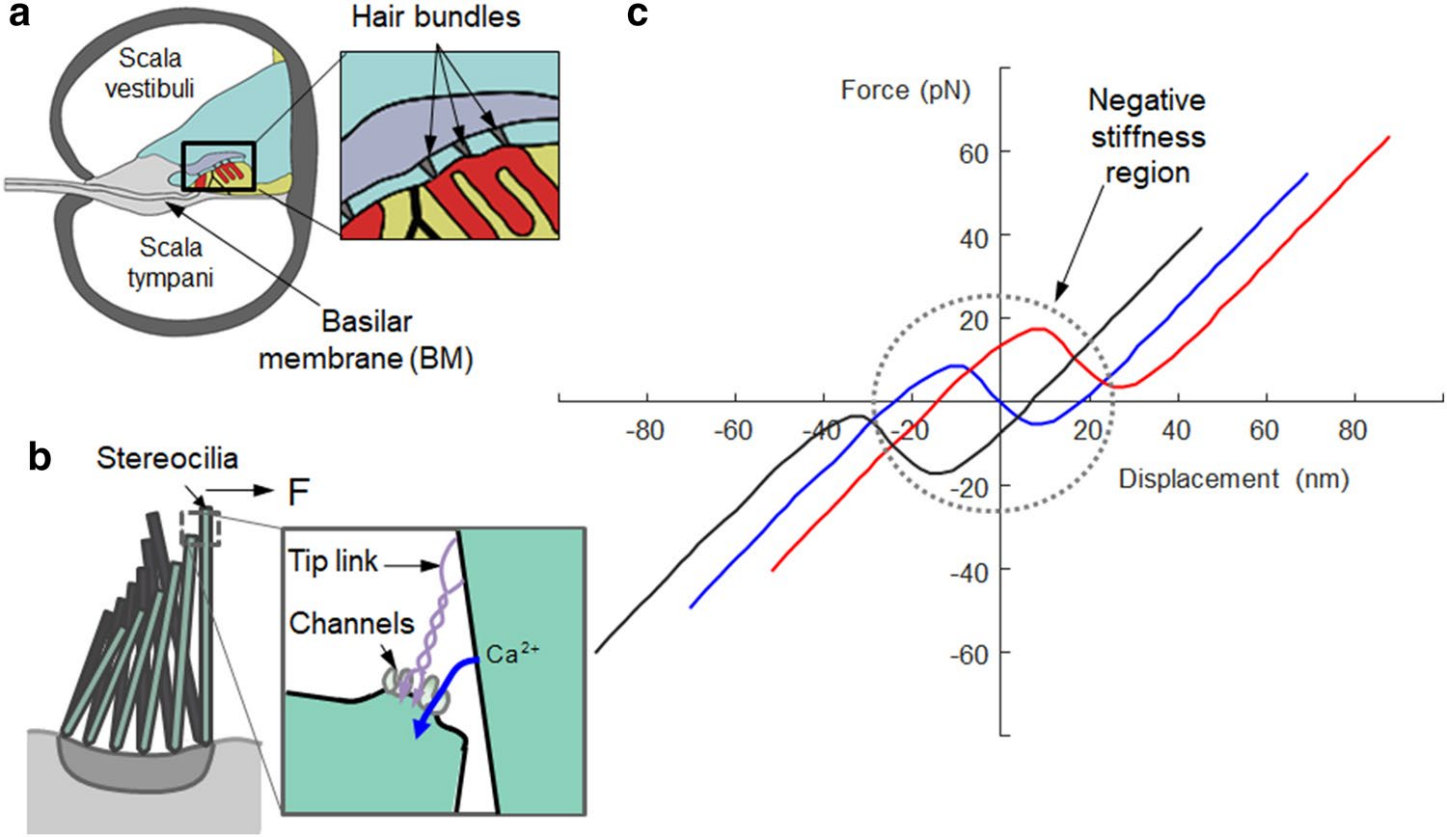
Bundle structures in cochlea outer hair cells: (**a**) a cross-sectional view of the cochlea in the auditory system; (**b**) densely bundled *stereocilia*; (**c**) its mechanical property featuring a negative stiffness region and adaptation ability observed in frog cochlea^6^.

The cochlea present inside the inner ear is one of the primary auditory organs in which sound is transduced from acoustic energy into an electrical signal. Its sensory receptors are called hair cells and feature densely bundled hair structures^1^. The primary function of the hair bundle structure is to send biologically induced electrical impulse signals to the brain in response to the vertical oscillation produced by the traveling wave propagation on the basilar membrane of the cochlea, as shown in Fig. 1a. Typically, the hair bundle structures of the cochlear outer hair cells in auditory systems consist of multiple tiny long cylinders (e.g., approximately 100 in an ear) called *Stereocilia* that lean on each other with tip links in the hair cell, as shown in Fig. 1b. The primary function of hair bundles originates from the dynamics of the tip link (elastic gating spring) connected to transduction channel gates, as shown in Fig. 1b. Apart from the restoring force produced by the bending stiffness, the tip links in the hair bundles deliver an external force to the transduction channel^2^. Furthermore, calcium ion (Ca2^+^) concentration plays an essential role in impulse signal transduction because the channel’s open probability is determined by the hair bundle displacement and calcium ion concentration. As illustrated in Fig. 1b, individual hair bundles protruding from the bottom surface of the hair cells oscillate within the fluid-filled cochlea. Typically, in mammals, including humans, one row of the inner hair cells is aligned along the length of the cochlea in parallel to three rows of the outer hair cells. *Stereocilia* of the inner hair cells are linearly arranged, whereas outer hair bundles are arranged in a *V*-shaped pattern. These different morphologies are likely to reflect the distinct functions of inner and outer hair cells. Outer hair cells can amplify small oscillations, and therefore, significantly enhance the sensitivity and dynamic range of hearing. In contrast, inner hair cells do not amplify but transmit electrical signals to the auditory-nerve fibers^3,4^.

As shown in Fig. 1c, negative stiffness is experimentally observed when measuring the mechanical properties of the hair bundle structures of a frog^5–7^. The dynamic oscillator with this nonlinearity is referred to as a bistable oscillator, and it has a unique double-well restoring force potential and provides three distinct operating regions depending on the input amplitude. This bistable oscillator may be excited to exhibit aperiodic or nonlinear vibrations between the two wells (i.e., the negative-stiffness region). Thus, numerous studies have attempted to harness the bistability featuring a snap-through (also known as buckling) action in various engineering applications, such as energy harvesting devices, acoustic transducers, and fluidic sensors^8–11^.

To date, it has been hypothesized that bistability is harnessed to amplify mechanical auditory stimuli in hair bundle structures. The sensitivity of hearing can be enhanced by the mechanoelectrical phenomenon observed in the outer hair bundle structures, which can amplify hair cell motion over broad frequency ranges. This high sensitivity is also hypothesized to exist because of the combination of an adaptation and a negative stiffness property inherent in the hair bundle structures^12^. Hence, even though a sinusoidal stimulus is applied in a specific frequency range, the transferred sound signals can be amplified owing to the mechanical adaptation ability that shifts the region of highest sensitivity toward the active operation range of the hair cell structure. Although the detailed mechanism by which mechanical energy is amplified by the nonlinearity of hair cells describes well^3,5^, some relevant studies have still attempted to further identify the amplification mechanism^13–15^. In this study, we mimic only its mechanical property featuring a negative stiffness region and adaptation ability. The primary objective of this study is thus to investigate the nonlinear dynamic behavior of a bistable oscillator with adaptive bistable stiffness, mimicking hair cell bundle structure in a frog cochlea, and aims to harness its bistable nonlinearity that features a negative stiffness region for broadband vibration applications.

## Mathematical formulation of hair cell bundle structure

**Figure 2:**
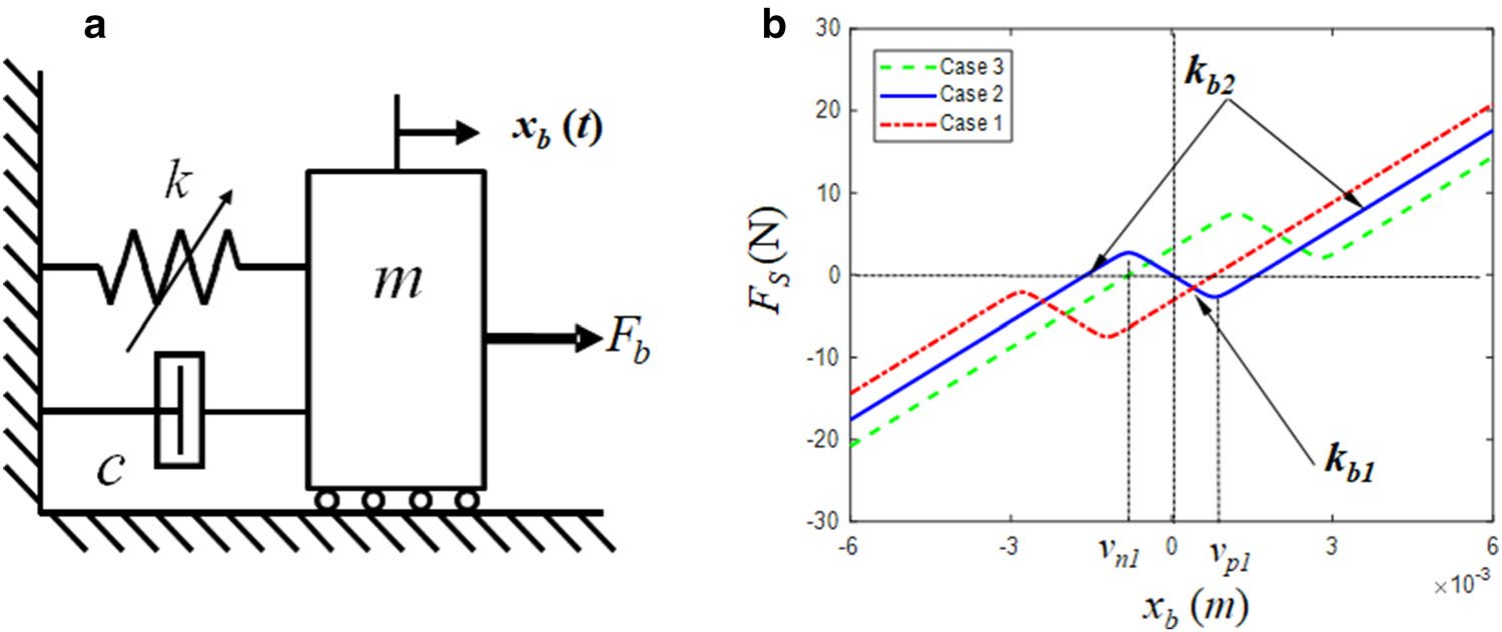
Hair-cell-bundle structure with nonlinear spring characteristics: (**a**) bilinear oscillator; (**b**) its bistable stiffness curves showing negative stiffness region: , Case 1; , Case 2 (baseline); , Case 3.

In this study, the nonlinear stiffness characteristic of the hair cell bundle structure is represented by the nonlinear spring element of a 1-DOF vibrating system (i.e., a bistable oscillator), as shown in Fig. 2a. The nonlinear stiffness function of the hair cell bundle structure includes two identical positive and one negative stiffness coefficient. The equation of motion for a 1-DOF vibrating system can then be formulated as follows

1 $$m\ddot{x}_{b} (t) + c\dot{x}_{b} (t) + F_{S} (x_{b} ) = F_{b} (t).$$

where $F_{S} (x)$ and $F_{b} \left( t \right)$ denote nonlinear spring and sinusoidal excitation forces, respectively. Similar to the stiffness curves displayed in Fig. 1c, the bistable nonlinearity is mathematically formulated using the hyperbolic tangent function to avoid instability due to the discontinuity of using the piecewise linear function. The effectiveness of the hyper tangent function for the sigmoid (*S*-shaped) function has been proved in many prior research^16^.

2a $$F_{S} \left( {x_{b} } \right) = F_{S1} \left( {x_{b} } \right) + F_{Spr} ,$$

2b $$F_{S1} \left( {x_{b} } \right) = k_{b1} x_{bpr} + \frac{1}{2}\sum\limits_{i = 2}^{{N_{\max } }} {\left( {k_{b\left( i \right)} - k_{{b\left( {i - 1} \right)}} } \right)\left( {F_{{bp\left( {i - 1} \right)}} - F_{{bn\left( {i - 1} \right)}} } \right)} ,$$

2c $$F_{bp\left( i \right)} = \left( {x_{bpr} - \nu_{p\left( i \right)} } \right)\left[ {tanh\left\{ {\sigma_{b} \left( {x_{bpr} - \nu_{p\left( i \right)} } \right)} \right\} + 1} \right],$$

2d $$F_{bn\left( i \right)} = \left( {x_{bpr} + \nu_{n\left( i \right)} } \right)\left[ {tanh\left\{ {\sigma_{b} \left( {x_{bpr} + \nu_{n\left( i \right)} } \right)} \right\} - 1} \right].$$

where the employed variables and symbols are as follows: *k*_*b*(*i*)_, such as *k*_*b*1_ and *k*_*b*2_, stiffness values for each stage; *x*_*bpr*_ = *x*_*b*_ − *x*_*pr*_; *x*_*pr*_, preload location; *F*_*S*_, total spring force; *F*_*S*1_, spring force without the preload; *F*_*Spr*_, preload; *F*_*bp*(*i*)_, spring force on the positive side; *F*_*bn*(*i*)_, spring force on the negative side; *ν*_*p*(*i*)_, transition displacement on the positive side; *ν*_*n*(*i*)_, transition displacement on the negative side; *σ*_*b*_, smoothing factor. Thus, arbitrary piecewise-type nonlinearities of the bistable stiffness profiles can be determined by employing their numerical values. Case 2 in Fig. 2b shows the symmetric-type bistable stiffness, calculated by substituting the relevant properties. Because the stiffness of the hair cell bundle structure in Fig. 1c is observed by microscale [Force ($10^{ - 12}$)/Displacement ($10^{ - 9}$)] and it is an extremely small value ill-suited for real engineering applications, it was scaled to the macro level (i.e., $10^{3}$), as listed in Table 1^11^. For different piecewise-type nonlinearities, multiple profiles can be produced using Eq. (2), as shown in Fig. 2b. In addition, to formulate each bistable stiffness curve, the smoothing factor *σ*_*b*_ is set as 5 × 10^3^. Table 2 lists the relevant properties to determine three cases: Cases 1, 2, and 3. For example, Case 2 reflects the symmetric characteristics; however, Cases 1 and 3, which mimic the adaptive bistable stiffness, are shifted from the origin and become asymmetric. Thus, all possible bistable stiffness profiles can be defined by employing the proper values for *F*_*Spr*_ and *x*_*pr*_ based on the symmetric case, such as in Case 2. For example, Table 2 lists the values for *F*_*Spr*_ and *x*_*pr*_ to determine three different cases of nonlinear stiffness profiles, as shown in Fig. 2b. The proof mass *m* = 0.015 kg; damping coefficient *c* = 51.6 N s m^−1^, assuming that the employed modal damping ratio, *ζ*, and natural frequency, *ω*_*n*_ (*f*_*n*_), are 5% and 516.4 rad s^−1^ (82.2 Hz) respectively. Additionally, the natural frequency *ω*_*n*_ is obtained using the positive stiffness value *k*_*b*2_ = 4 × 10^3^ N m^−1^.

**Table 1:** Properties of the bistable stiffness values for the symmetric case.

Property	Stage	Value
Bistable stiffness, *k*_*bi*_ (linearized in a piecewise manner) (N/m)	1	− 4 × 10^3^
	2	4 × 10^3^
Transition displacement on the positive side (*x*_*b*_ > 0), $\nu_{pi}$ (m)	1	8 × 10^−4^
	2	6 × 10^−3^
Transition displacement on the negative side (*x*_*b*_ < 0), $\nu_{ni}$ (m)	1	− 8 × 10^−4^
	2	− 6 × 10^−3^

**Table 2:** Properties of preload and their locations for each case.

Case	Preload, *F*_*Spr*_ (N)	Location, *x*_*pr*_ (m)
Case 1	− 4.8	− 2 × 10^−3^
Case 2 (Baseline)	0	0
Case 3	4.8	2 × 10^−3^

## Nonlinear frequency response analysis bistable oscillator

To investigate the system nonlinear responses, the harmonic balanced method (HBM) was implemented in this study based on the Galerkin schemes because all the information of the system responses including the stability conditions in both time and frequency domains under the steady state conditions is efficiently obtained by employing the HBM and it has been known as one of the powerful tools to analyze the strongly nonlinear stiffness system^16–19^. First, the system response *x*_*b*_(*t*) and the input *F*_*b*_(*t*) can be considered as follows:

3a $$x_{b} \left( t \right) = x_{m} + \sum\limits_{k = 1}^{{\eta N_{\max } }} {\left( {x_{ak} cos\frac{k}{\eta }\omega t + x_{bk} sin\frac{k}{\eta }\omega t} \right)} ,$$

3b $$F_{b} \left( t \right) = F_{m} + \sum\limits_{k = 1}^{{N_{\max } }} {F_{pk} cos\left( {k\omega_{p} t + \varphi_{pk} } \right)} .$$

where *x*_*m*_, *x*_*ak*_, and *x*_*bk*_ are the mean and alternating parts of the cosine and sine functions for the system responses, respectively; *F*_*m*_ is the average force (0.1 *N*); $\omega_{p}$ and $\varphi_{pk}$ are the excitation frequency and phase angle (in this study, 0), respectively; $\eta$ and *k* are the sub- and super-harmonic indices, respectively; *N*_max_ is the maximum number of harmonics correlated with the harmonic index of the HBM. *Fpk* is the magnitude of the sinusoidal input force and selected to be 7 *N* such that it can induce the chaotic interwell vibrations^8^; Assuming that the system is in a steady state, the Galerkin scheme in Eq. (1) is expressed as follows^20,21^.

4 $$- \omega^{2} m\underline{\underline{{\boldsymbol{\Im }}}} \underline{\underline{\mathbf{D}}}^{\prime \prime } \underline{{\mathbf{x}_{\mathbf{c}} }} + \omega c\underline{\underline{{\boldsymbol{\Im }}}} \underline{\underline{\mathbf{D}}}^{\prime } \underline{{\mathbf{x}_{\mathbf{c}} }} + \underline{{\mathbf{F}_{\mathbf{S}} }} \left( {\underline{{\mathbf{x}_{\mathbf{b}} }} } \right) - \underline{{\mathbf{F}_{\mathbf{b}} }} \left( \mathbf{t} \right) = \underline{\mathbf{0}} .$$

Here, its relevant terms are defined as follows.

5a,b $$\underline{{{\mathbf{x}}_{\mathbf{b}} }} \left( \mathbf{t} \right) = \underline{\underline{{\boldsymbol{\Im }}}} \underline{{{\mathbf{x}}_{\mathbf{c}} }} ,\;\underline{{{\mathbf{x}}_{\mathbf{b}} }} \left( \mathbf{t} \right) = \left[ {\begin{array}{*{20}c} {{\varvec{x}}_{{\varvec{b}}} \left( {t_{0} } \right)} & {{\varvec{x}}_{b} \left( {t_{1} } \right)} & \cdots & {{\varvec{x}}_{{\varvec{b}}} \left( {t_{m - 2} } \right)} & {{\varvec{x}}_{{\varvec{b}}} \left( {t_{m - 1} } \right)} \\ \end{array} } \right]^{{\text{T}}} ,$$

5c $$\underline{{{\mathbf{x}}_{{\mathbf{c}}} }} = \left[ {\begin{array}{*{20}c} {x_{m} } & {x_{a1} } & {x_{b1} } & \cdots & {x_{ak} } & {x_{bk} } & \cdots & {x_{{a\left( {\eta N_{\max } } \right)}} } & {x_{{b\left( {\eta N_{\max } } \right)}} } \\ \end{array} } \right]^{T} ,$$

5d $$\underline{\underline{{\boldsymbol{\Im }}}} = \left[ {\begin{array}{*{20}c} 1 & \cdots & {cos\left( {k\psi_{0} } \right)} & {sin\left( {k\psi_{0} } \right)} & \cdots \\ 1 & \cdots & {cos\left( {k\psi_{1} } \right)} & {sin\left( {k\psi_{1} } \right)} & \cdots \\ {} & \ddots & {} & {} & \ddots \\ 1 & \cdots & {cos\left( {k\psi_{N - 2} } \right)} & {sin\left( {k\psi_{N - 2} } \right)} & \cdots \\ 1 & \cdots & {cos\left( {k\psi_{N - 1} } \right)} & {sin\left( {k\psi_{N - 1} } \right)} & \cdots \\ \end{array} } \right],\;\underline{\underline{{\boldsymbol{\Im }}}}^{\prime } = \omega \underline{\underline{{\boldsymbol{\Im }}}} \underline{\underline{\mathbf{D}}}^{\prime } ,\;\underline{\underline{{\boldsymbol{\Im }}}}^{\prime \prime } = - \omega^{2} \underline{\underline{{\boldsymbol{\Im }}}} \underline{\underline{\mathbf{D}}}^{\prime \prime } ,$$

5e,f $$\underline{\underline{\mathbf{D}}}^{\prime } = \left[ {\begin{array}{*{20}c} 0 & {} & {} & {} \\ {} & \ddots & {} & {} \\ {} & {} & {\left[ {\begin{array}{*{20}c} 0 & k \\ { - k} & 0 \\ \end{array} } \right]} & {} \\ {} & {} & {} & \ddots \\ \end{array} } \right],\;\underline{\underline{\mathbf{D}}}^{\prime \prime } = \left[ {\begin{array}{*{20}c} 0 & {} & {} & {} \\ {} & \ddots & {} & {} \\ {} & {} & {\left[ {\begin{array}{*{20}c} {k^{2} } & 0 \\ 0 & {k^{2} } \\ \end{array} } \right]} & {} \\ {} & {} & {} & \ddots \\ \end{array} } \right].$$

Furthermore, its nonlinear and input functions are defined as follows.

6a,b $$\underline{{\mathbf{F}_{\mathbf{S}} }} \left( {\underline{{\mathbf{x}_{\mathbf{b}} }} } \right) = \underline{\underline{{\boldsymbol{\Im }}}} \underline{{\mathbf{F}_{{\mathbf{Sc}}} }} ,\;\underline{{\mathbf{F}_{\mathbf{b}} }} \left( \mathbf{t} \right) = \underline{\underline{{\boldsymbol{\Im }}}} \underline{{\mathbf{F}_{{\mathbf{bc}}} }} ,$$

6c $$\underline{{\mathbf{F}_{{\mathbf{Sc}}} }} = \left[ {\begin{array}{*{20}c} {F_{sm} } & {F_{sa1} } & {F_{sb1} } & \cdots & {F_{sa\left( l \right)} } & {F_{sb\left( l \right)} } & \cdots & {F_{{sa\left( {\eta N_{\max } } \right)}} } & {F_{{sb\left( {\eta N_{\max } } \right)}} } \\ \end{array} } \right]^{T} ,$$

6d $$\underline{{\mathbf{F}_{{\mathbf{bc}}} }} = \left[ {\begin{array}{*{20}c} {F_{m} } & {F_{a\left( 1 \right)} } & {F_{b\left( 1 \right)} } & \cdots & {F_{a\left( l \right)} } & {F_{b\left( l \right)} } & \cdots & {F_{{a\left( {\eta N_{\max } } \right)}} } & {F_{{b\left( {\eta N_{\max } } \right)}} } \\ \end{array} } \right]^{T} .$$

The relevant variables used are: $\varpi t = \psi$ and $\varpi = {\omega \mathord{\left/ {\vphantom {\omega {\omega_{n} }}} \right. \kern-0pt} {\omega_{n} }}$, the non-dimensionalized time scale, and the normalized frequency value with the natural frequency $\omega_{n}$; $T = \eta \tau$, is the concerned period with respect to $0 \le t < T$ → $0 \le \psi < {{2\pi } \mathord{\left/ {\vphantom {{2\pi } {\omega_{n} }}} \right. \kern-0pt} {\omega_{n} }}$; $\eta$ is a sub-harmonic index; $\tau$ is the period of the fundamental excitation; *k* and* l* represent the incremental index where $k = \omega_{n} , \, 2\omega_{n} , \, 3\omega_{n} \cdots$ and $l = 1, \, 2, \, 3 \cdots$. By employing the relationship between $\dot{x}\left( t \right) = \frac{dx}{{dt}} = \varpi \frac{dx}{{d\psi }} = \varpi x^{\prime}$ and $\ddot{x}\left( t \right) = \varpi^{2} x^{\prime\prime}$, the overall Galerkin scheme for the basic equation of Eq. (4) is expressed as follows:

7a,b $$\underline{\underline{{\boldsymbol{\Im }}}} \underline{{{\varvec{\Delta}}}} = \underline{\mathbf{0}} ,\;\underline{{{\varvec{\Delta}}}} = - \varpi^{2} m\underline{\underline{\mathbf{D}}}^{\prime \prime } \underline{{\mathbf{x}_{{\mathbf{bc}}} }} + \varpi c\underline{\underline{\mathbf{D}}}^{\prime } \underline{{\mathbf{x}_{{\mathbf{bc}}} }} + \underline{{\mathbf{F}_{{\mathbf{Sc}}} }} - \underline{{\mathbf{F}_{{\mathbf{bc}}} }} = \underline{\mathbf{0}} .$$

To determine the solutions of $\underline{{\mathbf{x}_{{\mathbf{bc}}} }}$ and $\varpi$ for each step, the Newton–Raphson method was implemented under the condition $\underline{{{\varvec{\Delta}}}} \to \underline{\mathbf{0}}$, where $\underline{{{\varvec{\Delta}}}}$ is considered a function of $\underline{{\mathbf{x}_{{\mathbf{bc}}} }}$ and $\varpi$, such as $\underline{{{\varvec{\Delta}}}} \left( {\underline{{\mathbf{x}_{{\mathbf{bc}}} }} ,\varpi } \right)$. Detailed information on the derivation and descriptions of the HBM can be found in previous studies^16^.

## Results and discussion

**Figure 3:**
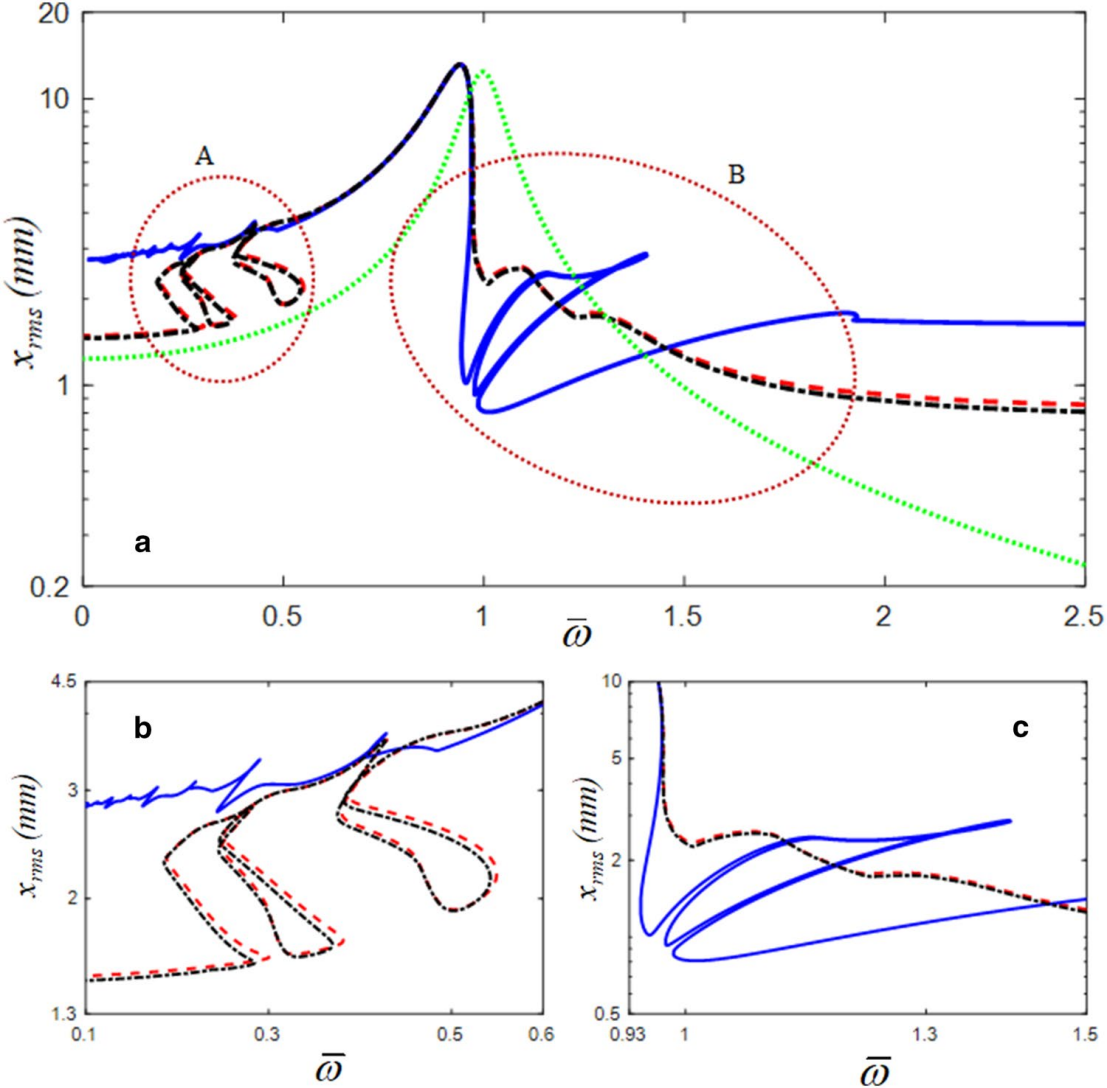
Nonlinear frequency responses with RMS values: (**a**) comparisons of HBM results with $\eta$ = 2 and *N*_max_ = 12 under three cases; (**b**) detail of A, super-harmonic regimes; (**c**) detail of B, sub-harmonic regimes: , Case 1; , Case 2 (baseline); , Case 3; , linear.

The HBM results with the root mean square (RMS) values of the displacement along with three different bistable stiffness curves are compared in Fig. 3, in which Cases 1 and 3 are asymmetric profiles, and Case 2 is a symmetric curve (a baseline for this study). Here, Cases 1 and 3 are shifted from the symmetric profile to the lower left and upper right directions, respectively, which corresponds to the mechanical adaptation capability. Cases 1 and 3 exhibit higher resonance values in the super-harmonic regimes than in the resonant regime of Case 2, as shown in Fig. 3b. However, Case 2 is highly affected by the sub-harmonic resonances compared with Cases 1 and 3, as shown in Fig. 3c. In addition, Cases 1 and 3 exhibit almost the same dynamic characteristics, whereas Case 2 shows significant differences in the super- and sub-harmonic areas, except for the primary resonance. To obtain the nonlinear responses in Fig. 3, the employed values of $\eta$ and *N*_max_ for the HBM are 2 and 12, respectively.

**Figure 4:**
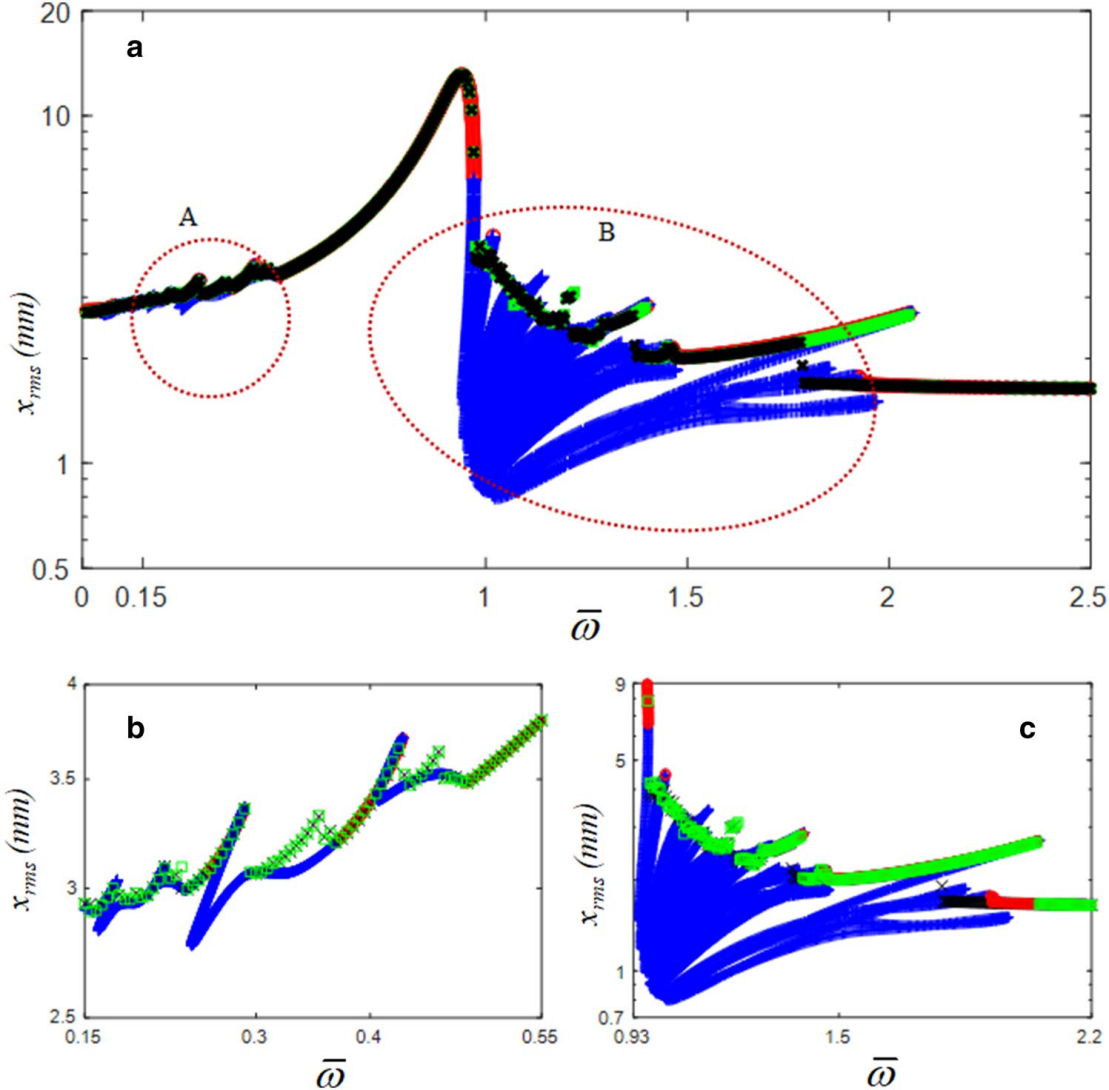
Comparisons of NS and HBM with $\eta$ = 6 and *N*_max_ = 12: (**a**) comparisons of HBM with NS; (**b**) super-harmonic regimes; (**c**) sub-harmonic regimes: , HBM results under stable conditions; +, HBM results under the unstable conditions; , NS result with frequency up-sweeping; ×, NS result with frequency down-sweeping.

Figure 4 shows a comparison of the numerical simulation (NS) and the HBM results. In this study, the modified Runge–Kutta method was employed to obtain the NS result^22^. To reveal the sub-harmonic responses in greater detail, the values for $\eta$ and *N*_max_ of the HBM were 6 and 12, respectively. A greater number of complex sub-harmonic responses could be obtained with an increase in the number of sub-harmonic indices $\eta$, as shown in Fig. 4. The red dotted circles indicated as (A) and (B) represent the super- and sub-harmonic regimes, respectively, and the stability conditions were determined using Hill’s method^23–25^. The details of the super- and sub-harmonic responses are shown in Fig. 4b and c. When the NS and HBM results were compared, the stable responses of the HBM correlated well with the NS results. However, the unstable response of the HBM was not correlated well because the unstable response is related to complicated system behaviors, such as quasi-periodic and chaotic phenomena. In addition, the differences between the NS and HBM results were due to different analytical processes. For example, NS is calculated based on the time domain integration using the initial conditions renewed at each prior and current step^22^. Thus, the NS can reflect all possible dynamic behaviors with time variations. However, the HBM determines its solutions efficiently by estimating the frequency and time domain information with the integer-based Fourier expansion, even though it cannot include all the possible time histories, such as transient responses^19–21,23,24^. Based on two different analytical approaches, the super- and sub-harmonic responses induced by bistable nonlinearities can be investigated in detail.

### Nonlinear dynamic behaviors in the super-harmonic regime

**Figure 5:**
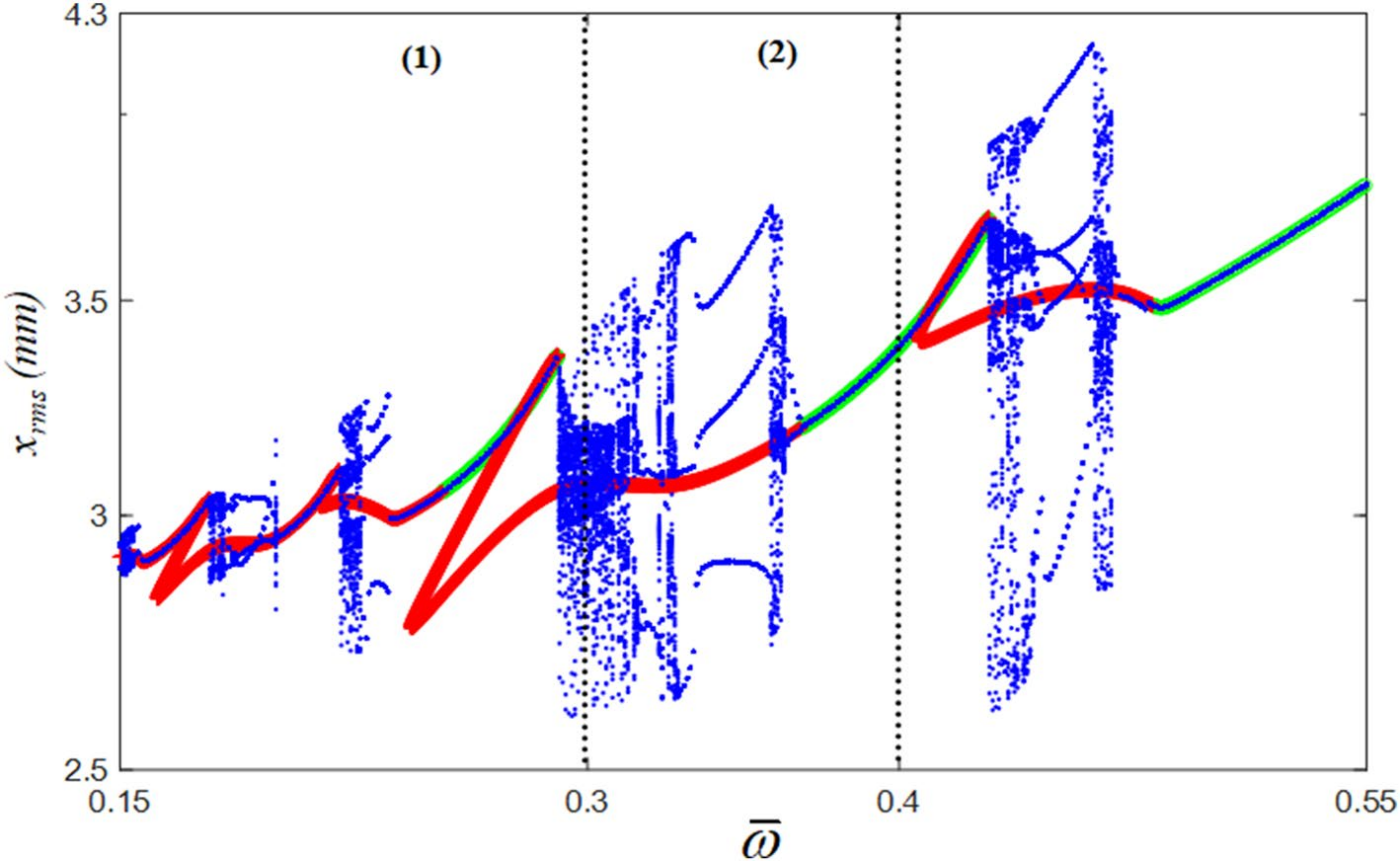
Bifurcation diagram projected in the super-harmonic regimes compared with HBM: , stable solutions of HBM; +, unstable solutions of HBM; , bifurcation diagram.

The nonlinear dynamic characteristics of the NS and HBM were compared with the bifurcations in the super-harmonic regime, as shown in Fig. 5. In general, the bifurcation is defined as the dramatic changes of the system responses under the small variation of system parameters. For example, $\varpi$ is considered as a parameter for the current system shown in Fig. 5. While the value $\varpi$ is changed from the lower to the higher ranges, some of areas around $\varpi$ = 0.3 show various solutions whenever each period is complete, which appears as scattered blue dots, as shown in Fig. 5. To calculate the bifurcations, the solutions of NS for each excitation frequency were obtained at the same orbital locations during 100 cycles of periodic motions after the transient responses were completely removed. When the stable solutions of the HBM are compared with those of the NS, they correlate well with each other, as shown in Fig. 5. However, unstable solutions of the HBM are closely related to bifurcation phenomena. By focusing on the area where the stability conditions change from unstable to stable (UTS), the UTS conditions reflect the bifurcation conditions well^18^. To analyze the dynamic responses in detail, the time histories can be reviewed in terms of two specific locations, $\varpi$ = 0.3 and $\varpi$ = 0.4, represented by (1) and (2), as shown in Fig. 5. For example, the system response at $\varpi$ = 0.3 shows the unstable conditions from the HBM, corresponding to the period constituting the bifurcation cascade obtained by NS.

**Figure 6:**
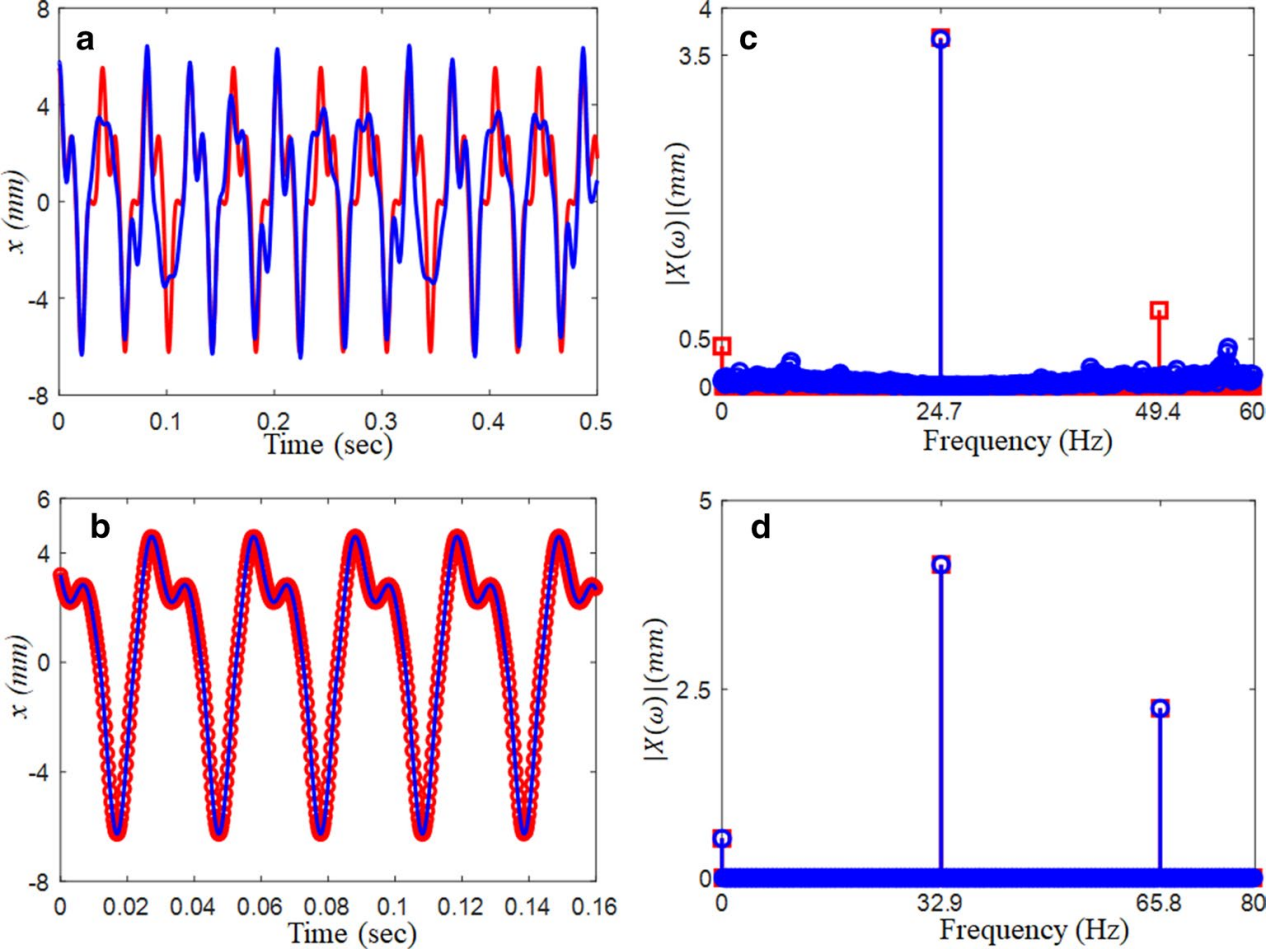
Comparison of the time histories between HBM ($\eta$ = 6 and *N*_max_ = 12) and NS at different excitation conditions: (**a**) time histories at $\varpi = 0.3$ (24.7 Hz); (**b**) time histories at $\varpi = 0.4$ (32.9 Hz). Key: , HBM; , NS., (**c**) FFT spectrum at $\varpi = 0.3$ (24.7 Hz); (**d**) FFT spectrum at $\varpi = 0.4$ (32.9 Hz): , HBM; , NS.

In this regime, the time histories exhibit significant complexities, as shown in Fig. 6a. When the time histories calculated by both the NS and HBM are compared, the NS results include several harmonic terms rather than the HBM because HBM is constructed using integer-based increment, as described earlier. In addition, the harmonic components in terms of magnitude are clearly observed in Fig. 6c. While the FFT results from the NS, as shown in Fig. 6c, demonstrate multiple harmonic terms, the HBM shows only fundamental and 2nd harmonic terms. Meanwhile, the system responses at $\varpi$ = 0.4 show good correlations between the two results between NS and HBM, as shown in Fig. 6b, because the dynamic behavior at this location is stable, which is also observed in the FFT spectrum shown in Fig. 6d.

**Figure 7:**
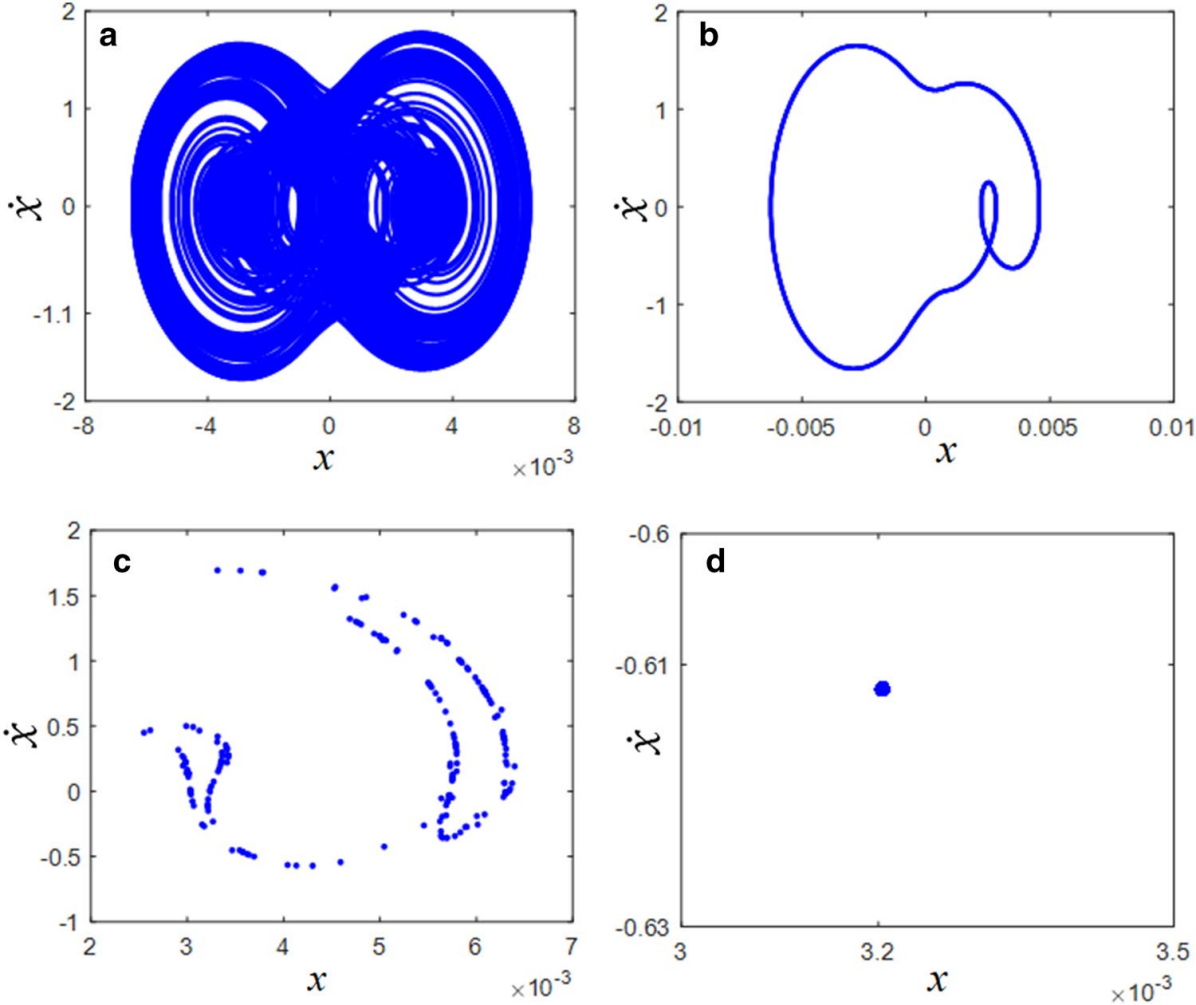
Comparisons of phase diagrams and Poincare maps in the super-harmonic regimes: (**a**) phase diagram at $\varpi = 0.3$; (**b**) phase diagram at $\varpi = 0.4$; (**c**) Poincare map at $\varpi = 0.3$; (**d**) Poincare map at $\varpi = 0.4$.

In addition, the complexities of the dynamic behaviors could be efficiently examined from the phase diagrams and Poincare maps. Figure 7 shows a comparison of the phase diagrams and Poincare maps at $\varpi$ = 0.3 and $\varpi$ = 0.4. For instance, the phase diagram at $\varpi$ = 0.3 includes more complex dynamic tracks, as shown in Fig. 7a. However, the phase diagram at $\varpi$ = 0.4 shows only one clear cycle. The Poincare map in Fig. 7c demonstrates scattered points. However, the Poincare map at $\varpi$ = 0.4 is concentrated at only one point.

### Nonlinear dynamic behaviors in the sub-harmonic regime

**Figure 8:**
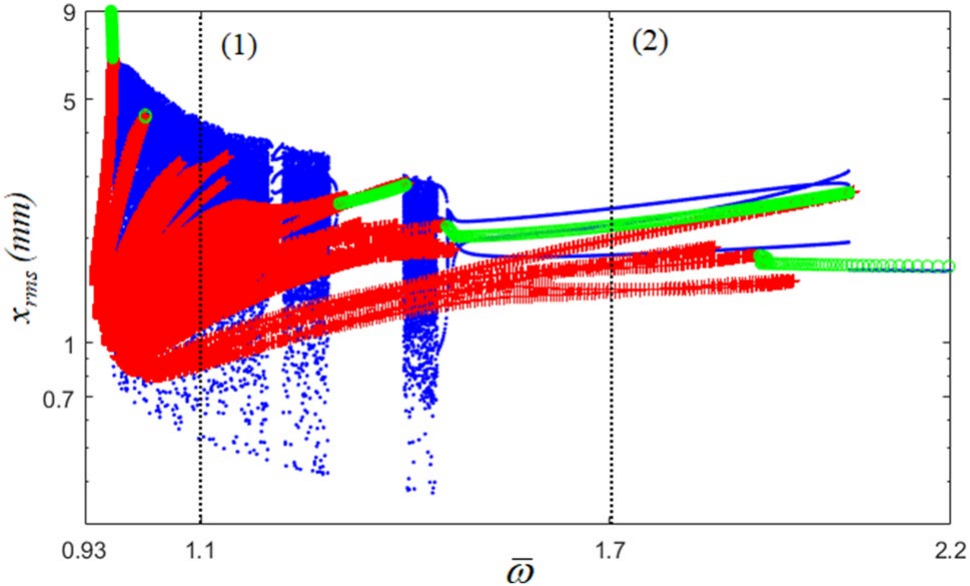
Bifurcation diagram projected in the sub-harmonic regimes compared with HBM: , stable solutions of HBM; +, unstable solutions of HBM; , bifurcation diagram.

**Figure 9:**
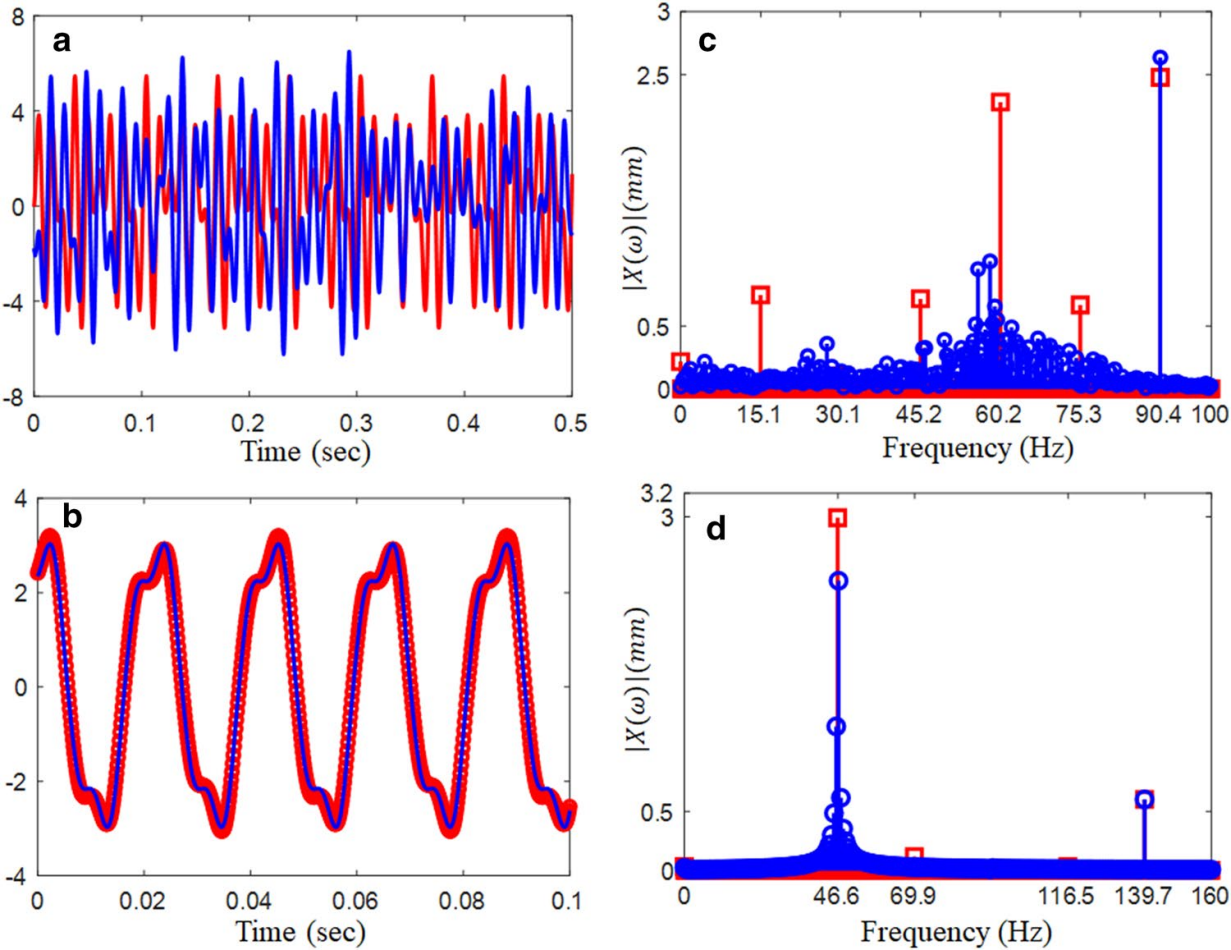
Comparison of the time histories between HBM ($\eta$ = 6 and *N*_max_ = 12) and NS at different excitation conditions: (**a**) time histories at $\varpi = 1.1$ (90.4 Hz); (**b**) time histories at $\varpi = 1.7$ (139.7 Hz): , HBM; , NS, (**c**) FFT spectrum at $\varpi = 1.1$ (90.4 Hz); (**d**) FFT spectrum at $\varpi = 1.7$ (139.7 Hz): , HBM; , NS.

The nonlinear dynamic behaviors in the sub-harmonic regime are shown in Fig. 8. The dynamic behaviors in region (1) are affected by more nonlinearities because the stability conditions from HBM are determined to be unstable, and this region demonstrates severe bifurcation effects. However, region (2) shows relatively less complexity because this region pertains to the stable dynamic conditions determined by the HBM, even though the system responses in this region show a period-doubling effect. As shown in Fig. 9, the dynamic responses from the NS and HBM are compared based on the time histories. For example, Fig. 9a compares the time histories at $\varpi$ = 1.1, marked as (1), in which the results from both NS and HBM are not well correlated. This is the same reason as that observed in super-harmonic regions, implying that the system is affected by higher nonlinearities in region (1) than in region (2). With respect to these unstable conditions, the FFT spectrum also reflects complex nonlinearities, as observed in Fig. 9c. The FFT results from the HBM show the fundamental and relevant subharmonic terms, whereas those of the NS include various harmonic terms. The nonlinear dynamic behavior is more predictable in region (2) than in region (1), but its responses still show that their motions are affected by the period-doubling conditions, even though the HBM is determined to be stable, as shown in Fig. 8. Corresponding to this, the time histories from both the NS and HBM are nearly correlated, as shown in Fig. 9b and d. However, the FFT results of the NS include various harmonic terms around at ${\varpi \mathord{\left/ {\vphantom {\varpi 3}} \right. \kern-0pt} 3}$ (= 46.6 Hz) because its region is still affected by bifurcation, as shown in the marked area (2) of Fig. 8.

**Figure 10:**
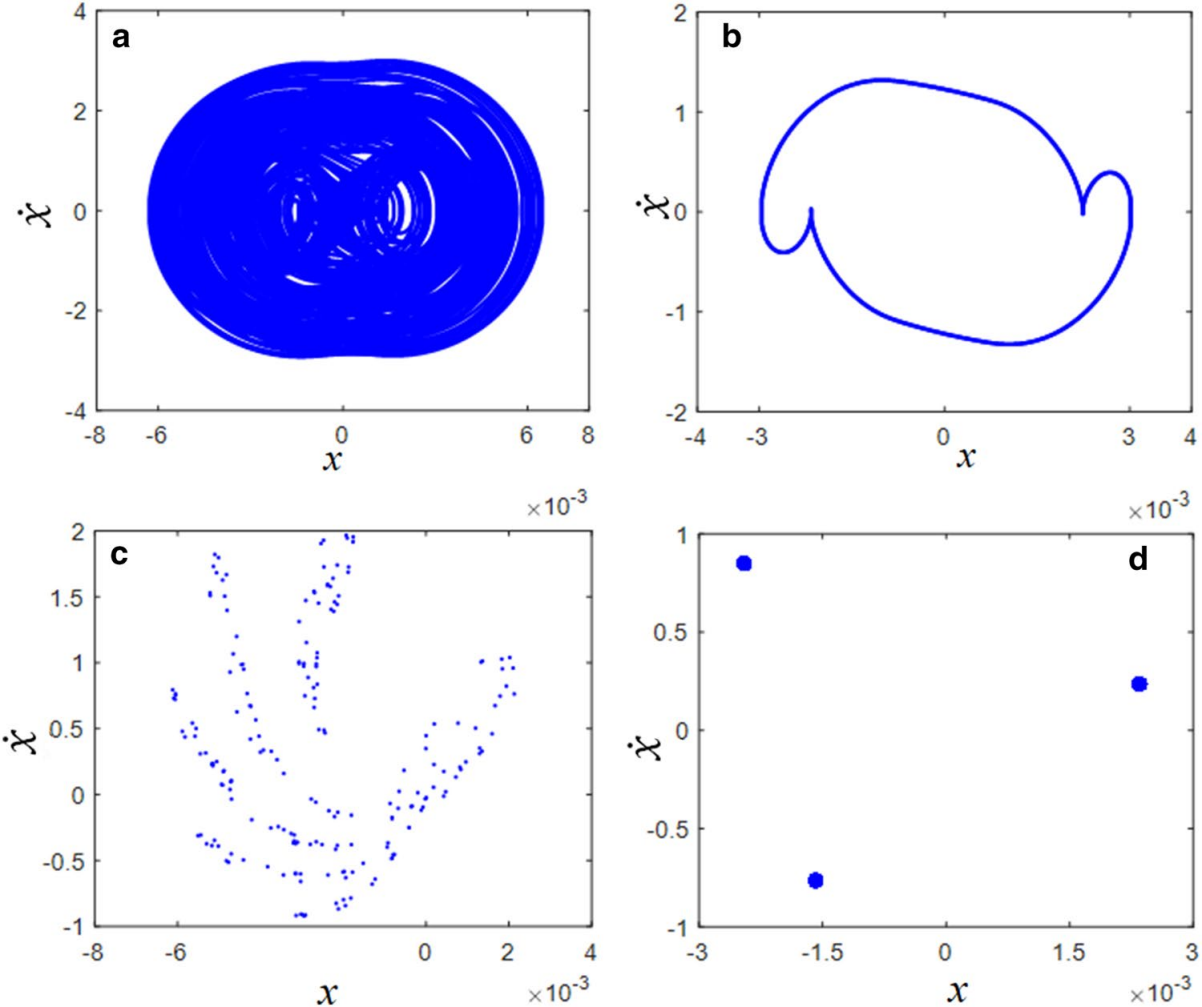
Comparisons of phase diagrams and Poincare maps in the sub-harmonic regimes: (**a**) phase diagram at $\varpi = 1.1$; (**b**) phase diagram at $\varpi = 1.7$; (**c**) Poincare map at $\varpi = 1.1$; (**d**) Poincare map at $\varpi = 1.7$.

The dynamic characteristics of the phase diagrams and Poincaré maps are shown in Fig. 10. Figure 10a and c show the dynamic behaviors of region (1), which are significantly affected by complex responses, as shown in Fig. 8. Meanwhile, Fig. 10b and d effectively reflect the dynamic motions in region (2) with one line of the track; however, the Poincare map shows that the periodic motions are not concentrated at one point because their responses are affected by the period-doubling bifurcation, as seen in Fig. 8.

**Figure 11:**
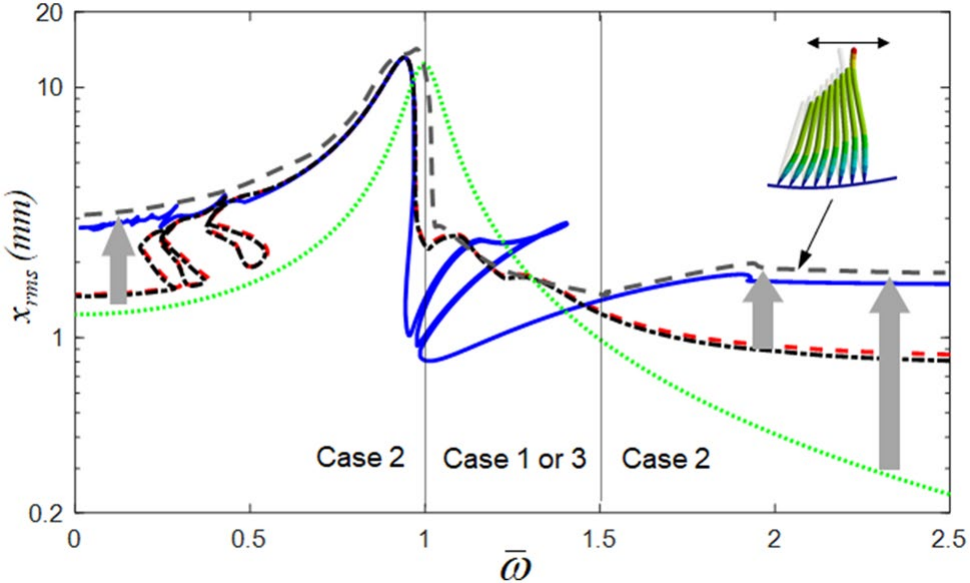
Proposed frequency response function (gray dashed line) tuned by adaptively changing the bistable stiffness.

Based on the nonlinear frequency response analysis, we propose the desired frequency response function (gray dashed line) tuned by adaptively changing the bistable stiffness, as shown in Fig. 11. Before resonance, the nonlinear stiffness for Case 2 was used to increase the magnitude (i.e., higher sensitivity) and avoid the super-harmonic response in Case 1 (or Case 3). After resonance, the nonlinear stiffness was adaptively shifted into Case 1 (or Case 3) to prevent the sub-harmonic response in Case 1. This nonlinear stiffness was recovered in Case 2 to increase the magnitude ratio (i.e., higher sensitivity) beyond $\overline{\omega } > 1.5$. Consequently, the frequency response function can be nearly uniformed after resonance (i.e., broadband)^26,27^. Once we fabricate a prototype that mimics the adaptive bistable stiffness of the hair cell bundle, this new adaptive structure enables the development of metamaterial-like broadband vibration applications, such as vibration-based energy harvesters, despite some technical issues that need to be further examined.

## Conclusions

In this study, the dynamic behavior of bistable oscillator with adaptive bistable stiffness, which is similar to the hair cell bundle structure in a frog cochlea, was successfully investigated. The main contributions of this study are summarized as follows.

- First, the bistable nonlinearity using the hyperbolic tangent function was effective to avoid instability induced when the discontinuity is connected with the piecewise linear functions when it combines with the harmonic balance method.
- Secondly, we investigated all possible nonlinear dynamic characteristics of bistable oscillator by examining the nonlinear frequency responses, phase diagrams, and Poincare maps.
- Lastly, we report for the first time that it is possible to achieve a new means of producing broadband (uniform, flat) frequency response functions by mimicking the adaptive bistable stiffness of hair cell bundles.

Overall, it is necessary to implement the proposed bistable oscillator, primarily focusing with adaptive stiffness switching mechanism although our study provides the initial information through the simulation for designing broadband vibration applications such as vibration-based energy harvesters. The adaptive bistable stiffness will be explored by combining different preload adjustment mechanisms with smart-material-based actuators. For the future research direction, we will continue to address some ongoing issues. In particular, further studies such as in-depth bifurcation analysis by mapping the Floquet multipliers are required to complete the nonlinear frequency–response analysis.

## Data Availability

The datasets used and/or analyzed during the current study are available from the corresponding author upon reasonable request.
